# Fecal IgA, Antigen Absorption, and Gut Microbiome Composition Are Associated With Food Antigen Sensitization in Genetically Susceptible Mice

**DOI:** 10.3389/fimmu.2020.599637

**Published:** 2021-01-19

**Authors:** Johanna M. Smeekens, Brandi T. Johnson-Weaver, Andrew L. Hinton, M. Andrea Azcarate-Peril, Timothy P. Moran, Robert M. Immormino, Janelle R. Kesselring, Erin C. Steinbach, Kelly A. Orgel, Herman F. Staats, A. Wesley Burks, Peter J. Mucha, Martin T. Ferris, Michael D. Kulis

**Affiliations:** ^1^ Department of Pediatrics, Division of Rheumatology, Allergy and Immunology, School of Medicine, University of North Carolina, Chapel Hill, NC, United States; ^2^ UNC Food Allergy Initiative, School of Medicine, University of North Carolina, Chapel Hill, NC, United States; ^3^ Department of Pathology, Duke University School of Medicine, Durham, NC, United States; ^4^ Curriculum in Bioinformatics and Computational Biology, University of North Carolina, Chapel Hill, NC, United States; ^5^ Department of Medicine, Division of Gastroenterology and Hepatology, University of North Carolina, Chapel Hill, NC, United States; ^6^ UNC Microbiome Core, Center for Gastrointestinal Biology and Disease, University of North Carolina, Chapel Hill, NC, United States; ^7^ Duke Human Vaccine Institute, Duke University School of Medicine, Durham, NC, United States; ^8^ Department of Immunology, Duke University School of Medicine, Durham, NC, United States; ^9^ Department of Mathematics and Department of Applied Physical Sciences, University of North Carolina, Chapel Hill, NC, United States; ^10^ Department of Genetics, School of Medicine, University of North Carolina, Chapel Hill, NC, United States

**Keywords:** food allergy, peanut allergy, IgE, microbiome, Tfh cells, fecal IgA, antigen absorption

## Abstract

Food allergy is a potentially fatal disease affecting 8% of children and has become increasingly common in the past two decades. Despite the prevalence and severe nature of the disease, the mechanisms underlying sensitization remain to be further elucidated. The Collaborative Cross is a genetically diverse panel of inbred mice that were specifically developed to study the influence of genetics on complex diseases. Using this panel of mouse strains, we previously demonstrated CC027/GeniUnc mice, but not C3H/HeJ mice, develop peanut allergy after oral exposure to peanut in the absence of a Th2-skewing adjuvant. Here, we investigated factors associated with sensitization in CC027/GeniUnc mice following oral exposure to peanut, walnut, milk, or egg. CC027/GeniUnc mice mounted antigen-specific IgE responses to peanut, walnut and egg, but not milk, while C3H/HeJ mice were not sensitized to any antigen. Naïve CC027/GeniUnc mice had markedly lower total fecal IgA compared to C3H/HeJ, which was accompanied by stark differences in gut microbiome composition. Sensitized CC027/GeniUnc mice had significantly fewer CD3^+^ T cells but higher numbers of CXCR5^+^ B cells and T follicular helper cells in the mesenteric lymph nodes compared to C3H/HeJ mice, which is consistent with their relative immunoglobulin production. After oral challenge to the corresponding food, peanut- and walnut-sensitized CC027/GeniUnc mice experienced anaphylaxis, whereas mice exposed to milk and egg did not. Ara h 2 was detected in serum collected post-challenge from peanut-sensitized mice, indicating increased absorption of this allergen, while Bos d 5 and Gal d 2 were not detected in mice exposed to milk and egg, respectively. Machine learning on the change in gut microbiome composition as a result of food protein exposure identified a unique signature in CC027/GeniUnc mice that experienced anaphylaxis, including the depletion of *Akkermansia*. Overall, these results demonstrate several factors associated with enteral sensitization in CC027/GeniUnc mice, including diminished total fecal IgA, increased allergen absorption and altered gut microbiome composition. Furthermore, peanuts and tree nuts may have inherent properties distinct from milk and eggs that contribute to allergy.

## Introduction

Food allergy is a potentially fatal disease that has increased in prevalence in the past two decades, now affecting an estimated 4%–8% of children and 10% of adults (1, 2). The most common foods to trigger allergic reactions are peanuts, tree nuts, milk, egg, soy, shellfish, fish, and wheat. Even trace amounts of an allergen can elicit a severe anaphylactic reaction. Therefore, allergic individuals must strictly avoid the offending allergen in order to prevent accidental exposure and reactions. As a result, the quality of life of allergic individuals and their caretakers is diminished (3). Of the eight most common food allergens, reactions to peanuts and tree nuts are most severe and account for 70%–90% of fatal allergic reactions (4, 5). Some food allergies, such as milk and egg, are often outgrown; however, peanut and tree nut allergies are only outgrown in an estimated 10% of individuals (6). The underlying characteristics and mechanisms that make peanut and tree nut allergies longer lasting and more severe are widely unknown.

Typically, oral ingestion of antigen leads to immunologic suppression of immune responses to the antigen, a process termed oral tolerance. These active processes in the gut prevent harmful immune responses to dietary antigens and commensal bacteria, while protecting against pathogens and toxins (7). CD4^+^CD25^+^Foxp3^+^ regulatory T cells (Tregs) are necessary for oral tolerance (8). CD103^+^ dendritic cells capture antigen in the lamina propria and then migrate to the mesenteric lymph nodes (MLNs) where they induce differentiation of naïve T cells to Tregs. These antigen-specific Tregs then migrate back to the lamina propria and expand (9). Failure in oral tolerance mechanisms to dietary antigens leads to T helper type 2 (Th2) immune responses, resulting in food allergy.

Secretory IgA in the gut also plays a crucial role in tolerance. IgA acts as a neutralizing antibody on epithelial surfaces and is hypothesized to prevent allergic immune responses by immune exclusion of luminal food proteins (10). Secretory IgA also regulates commensal bacteria composition in the gut, which in turn prevents uptake of toxins and pathogens (11). Altered microbiota composition is associated with oral tolerance failure and development of food allergy (12), and previous studies have demonstrated that several microbiota species, including the *Clostridia* class, are related to the mitigation of food allergy in mouse studies (13). Overall, the gut is a dynamic environment that involves regulation of microbiota by secretory IgA, which in turn can induce Treg production, leading to oral tolerance.

Murine models provide a platform to investigate the underlying mechanisms that lead to sensitization to food antigens. The Collaborative Cross is a genetically diverse panel of inbred mice that were specifically developed to study the influence of genetics on complex diseases (14, 15). Previously, we screened strains from the Collaborative Cross to identify an orally reactive model of peanut allergy (16). Specifically, CC027/GeniUnc mice were orally sensitized to peanut in the presence of cholera toxin and reacted on oral challenge. These mice had fewer Tregs and more intestinal mast cells compared to C3H/HeJ and C57BL/6J mice. Additionally, the CC027/GeniUnc mice could be sensitized to peanut in the absence of an adjuvant. Here, we aimed to explore the mechanisms of gut sensitization in this adjuvant-free model through exposure to peanut, walnut, milk, or egg, with a focus on fecal IgA, antigen absorption and gut microbiome. We utilized the C3H/HeJ strain as a comparator to CC027/GeniUnc because they are known to produce high quantities of IgE and are Th2-skewed, and are therefore used as a standard model of food allergy in the field (17–20).

## Materials and Methods

### Mice

CC027/GeniUnc mice were obtained from the UNC Systems Genetics Core Facility. A colony of C3H/HeJ mice originally obtained from the Jackson Labs was kept in the same facility where the CC027/GeniUnc mice were born, to standardize their environments. Mice were received for experiments starting at 4–6 weeks of age. Mice were kept on a 12:12 light:dark cycle and fed standard chow free of peanut, walnut, milk, and egg ingredients. All animal experiments were approved by the Institutional Animal Care and Use Committee at the University of North Carolina at Chapel Hill under protocol 17-286.

### Peanut, Walnut, Milk, and Egg Protein Extractions

Protein extractions were performed as reported previously (21). Briefly, proteins were extracted from roasted, defatted peanut flour (Golden Peanut, Alpharetta, GA), roasted, defatted walnut flour (Holmquist Hazelnut Orchards, Lynden, WA), nonfat dry milk powder (The Milky Whey, Missoula, MT) or egg white powder (Deb El Foods, Elizabethport, NJ) in PBS (supplemented with 1 M NaCl for peanut and walnut extractions). Protein concentrations were measured by BCA (Pierce, Waltham, MA) and extracts were determined to contain all major allergens by SDS-PAGE gel.

### Sensitization and Oral Food Challenges

Mice were exposed to food protein by oral gavage once per week for 4 weeks as follows: peanut and egg (2 mg the first 3 weeks, 5 mg the final week), walnut and milk (2 mg all 4 weeks). The following week, fecal pellets and serum were collected from mice. Mice were then challenged to the corresponding food *via* oral gavage (peanut and egg: 10 mg; milk and walnut: 5 mg). Core body temperatures were recorded every 15 min for 1 h post-challenge using a rectal probe (Physitemp, Clifton, NJ). Anaphylaxis was defined as a greater than 3°C decrease in body temperature. Mice were rested for several weeks, re-challenged and serum was collected 30 min post-challenge for allergen quantification. Several weeks after challenges, mice were euthanized for MLN isolation.

Food protein exposure resulted in 10 strain-food protein groups of mice: CC027-peanut, CC027-walnut, CC027-milk, CC027-egg, CC027-PBS, C3H-peanut, C3H-walnut, C3H-milk, C3H-egg, and C3H-PBS.

### Antigen-Specific IgE, IgG1, and IgG2a ELISAs

Antigen-specific IgE, IgG1 and IgG2a were quantified by ELISA as described previously (21). Briefly, 96-well plates were coated with 20 µg/ml peanut, walnut, milk, or egg extract for samples, or 20 µg/ml HSA-DNP (Millipore Sigma, St. Louis, MO) for standard curves, and blocked with 2% BSA in PBS-0.5% Tween. Samples were diluted 1:100 for IgE, 1:20,000 for IgG1, and 1:1,000 for IgG2a. Standard curves were prepared using mouse IgE anti-DNP, IgG1 anti-DNP or IgG2a anti-DNP (Accurate Chemicals, Westbury, NY) ranging from 0.002–2 µg/ml. The following antibodies were added to IgE plates in succession: 0.5 µg/ml sheep IgG anti-mouse IgE (The Binding Site, Birmingham, UK), 0.5 µg/ml biotinylated donkey anti-sheep IgG (Accurate Chemicals), and 0.5 µg/ml NeutrAvidin-HRP (Pierce Biotechnology, Rockford, IL). For IgG1 and IgG2a plates, HRP goat anti-mouse IgG1 (1:40,000, Southern Biotech, Birmingham, AL) or IgG2a (1:20,000, Southern Biotech) were added to plates. All plates were developed with TMB (SeraCare, Milford, MA), stopped with 1% HCl (SeraCare), and read at 450 nm using a spectrophotometer (BioTek Instruments, Winooski, VT).

### Allergen ELISAs (Ara h 2, Gal d 2, Bos d 5)

Serum collected 30 min post-challenge was diluted 1:10 and Ara h 2, Gal d 2, and Bos d 5 were quantified *via* ELISA according to the manufacturer’s instructions (Indoor Biotechnologies, Charlottesville, VA).

### Fecal IgA and IgG ELISAs

Fecal pellets were collected before and after the 4-week food protein exposure period, snap-frozen in liquid nitrogen, and stored at −80°C until analysis. Fecal pellets were resuspended in protein extraction buffer [10% goat serum (Fisher Scientific, Waltham, MA) in PBS] at a ratio of 10 mg per 100 µl, and vortexed for 20 min to disrupt pellets. Extracted samples were centrifuged for 10 min at 13,000 x g, and supernatants were collected for subsequent analysis.

ELISA was used to determine total IgG and IgA antibody concentrations present in mouse fecal extracts collected before and after the exposure period. Maxisorp 384-well black plates were coated with unlabeled goat anti-mouse IgG or IgA capture antibodies (Southern Biotech) at 5 μg/ml in carbonate/bicarbonate buffer and incubated at 4°C overnight. A similar ELISA method was used to detect allergen-specific IgG and IgA in fecal samples as described for total IgG and IgA ELISA with minor modifications. Maxisorp 384-well plates were coated with an individual allergen at a concentration of 2 μg/ml. Plates were blocked with non-fat dry milk blocking buffer for 2 h at room temperature. After blocking, ELISA plates were washed four times using PBS/0.1% Tween 20. Fecal samples and purified mouse IgG and IgA positive control standards were diluted two folds in complete sample diluent. The starting dilution for fecal samples was 1:8 and the starting concentration for the IgG and IgA standards was 1,000 ng/ml. Diluted samples and standards were added to the ELISA plates and incubated overnight at 4°C. After incubation, plates were washed to remove unbound antibodies prior to adding AP-labeled goat anti-mouse IgG or IgA secondary antibodies at a 1:8,000 dilution. Secondary antibodies were incubated at room temperature for 2 h. The fluorescent-based AttoPhos substrate system (Promega; Madison, WI) was used to develop the plates by incubating at room temperature for 15 min. Relative light units (RLU) for each sample were measured using a Biotek Synergy 2 plate reader (Winooski, VT).

The total IgG and IgA concentration for each fecal sample was determined using a standard curve calculator. The RLU values for the IgG and IgA standards and corresponding ng/ml concentration were used to develop a standard curve in GraphPad PRISM (San Diego, CA). The RLU values that fell within an acceptable range of the standard curves (80%–120% standard recovery) were used to interpolate an ng/ml concentration for each sample. The interpolated values for each sample were multiplied by the sample dilution factor. The final ng/ml concentration for each sample is the average of the interpolated values multiplied by the sample dilution factor.

The end-point titer was determined by the last sample dilution that provided a positive RLU value three-times greater than a naive reference sample at the same dilution as the sample. End-point titers are reported as geometric mean titers (GMTs).

### Mesenteric Lymph Node Analysis

MLNs were harvested, and lymphocytes were isolated from a subset of allergen-exposed mice. MLNs were homogenized and passed through a 70 µm strainer to obtain a single cell suspension and centrifuged at 1,500 rpm for 10 min at room temperature. Lymphocytes from MLNs were washed once with RPMI and centrifuged at 1,500 rpm for 10 min. Lymphocytes were resuspended in RPMI and counted using a hemocytometer.

Flow cytometric analysis of MLNs was performed as previously described (22). Briefly, cells were resuspended in FACS buffer (2 mM EDTA and 0.5% BSA in PBS) and incubated with anti-mouse CD16/CD32 (2.4G2) and 5% rat serum for 5 min to block Fc receptors. Cells were then incubated with fluorochrome-conjugated antibodies against murine CD3ϵ (145-2C11), CD4 (GK1.5), CD19 (6D5), CXCR5 (L138D7), and PD-1 (29F.1A12) for 30 min on ice. Cells were also concurrently stained with Zombie Aqua (BioLegend, San Diego, CA) for live cell/dead cell discrimination. Flow cytometry data were acquired with a four-laser LSRII (BD Biosciences, San Jose, CA) and analyzed using FlowJo (Ashland, OR) software. Only single cells were analyzed. Tfh cells were identified as CXCR5^+^PD-1^+^CD4^+^ T cells. All antibodies were purchased from BioLegend or BD Biosciences.

### Microbiome Analysis

Fecal pellets were collected before and after the 4-week food protein exposure period, snap-frozen in liquid nitrogen, and stored at −80°C until analysis.

#### DNA Isolation

DNA isolation was performed as previously described (23–25). Mouse stool samples were transferred to a 2 ml tube containing 200 mg of ≤106 μm glass beads (Sigma, St. Louis, MO) and 0.5 ml of Qiagen PM1 buffer (Valencia, CA). Bead beating was performed for 5 min in a Qiagen TissueLyser II at 30Hz. After a 5-min centrifugation, 0.45 ml of supernatants were aspirated and transferred to a new tube containing 0.15 ml of Qiagen IRS solution. The suspension was incubated at 4°C overnight. After a brief centrifugation, supernatants were aspirated and transferred to deep well plates containing 0.45 ml of Qiagen binding buffer supplemented with Qiagen ClearMag Beads. DNA was purified using the automated KingFisher™ Flex Purification System and eluted in DNase-free water.

#### 16S rRNA Amplicon Sequencing

Barcoding and library preparation were carried out as described (23, 24, 26). A total of 12.5 ng of DNA was amplified using universal primers targeting the V4 region of the bacterial 16S rRNA gene. Primer sequences contained overhang adapters appended to the 5’ end of each primer for compatibility with Illumina sequencing platform (27). The complete sequences of the primers were:

515F - 5’ TCGTCGGCAGCGTCAGATGTGTATAAGAGACAGGTGCCAGCMGCCGCGGTAA 3’

806R - 5’GTCTCGTGGGCTCGGAGATGTGTATAAGAGACAGGGACTACHVGGGTWTCTAAT 3’.

Master mixes contained 12.5 ng of total DNA, 0.5 µM of each primer and 2× KAPA HiFi HotStart ReadyMix (KAPA Biosystems, Wilmington, MA). The thermal profile for the amplification of each sample had an initial denaturing step at 95°C for 3 min, followed by a cycling of denaturing of 95°C for 30 s, annealing at 55°C for 30 s and a 30-s extension at 72°C (25 cycles), a 5 min extension at 72°C and a final hold at 4°C. Each 16S rRNA gene amplicon was purified using the AMPure XP reagent (Beckman Coulter, Indianapolis, IN). In the next step each sample was amplified using a limited cycle PCR program, adding Illumina sequencing adapters and dual‐index barcodes [index 1(i7) and index 2(i5), Illumina, San Diego, CA] to the amplicon target. The thermal profile for the amplification of each sample had an initial denaturing step at 95°C for 3 min, followed by a denaturing cycle of 95°C for 30 s, annealing at 55°C for 30 s and a 30-s extension at 72°C (8 cycles), a 5 min extension at 72°C and a final hold at 4°C. The final libraries were again purified using the AMPure XP reagent (Beckman Coulter), quantified and normalized prior to pooling. The DNA library pool was then denatured with NaOH, diluted with hybridization buffer and heat denatured before loading on the MiSeq reagent cartridge (Illumina) and on the MiSeq instrument (Illumina). Automated cluster generation and paired-end sequencing with dual reads were performed according to the manufacturer’s instructions.

#### Bioinformatics Analysis

Sequencing output from the Illumina MiSeq platform were converted to fastq format and demultiplexed using Illumina Bcl2Fastq 2.18.0.12. The resulting paired-end reads were processed using QIIME 2 2018.11 (28). Index and linker primer sequences were trimmed using the QIIME 2 invocation of cutadapt. The resulting paired-end reads were processed with DADA2 through QIIME 2 including merging paired ends, quality filtering, error correction, and chimera detection (29). Amplicon sequencing units from DADA2 were assigned taxonomic identifiers with respect to Greengenes release 13_08 using the QIIME 2 q2-featureclassifier (30). All sequencing data has been submitted to the NCBI repository and can be accessed *via* the following accession numbers: PRJNA674375.

#### Compositional Data Analysis

Compositional data analyses were performed on taxonomic count data from the ASV table after imputing values to replace zeroes. The taxa count matrices of the n = 78 samples and P = 230 taxa of the pre-exposure, **X**∈ℝ^n×P^, and post-exposure, **Y**∈ℝ^n×P^, gut microbiome profiles were processed as follows, respecting the compositional nature of the data (31, 32). First, both matrices were row-sum normalized, **X**′ = C[**X**], **Y**′ = C[**Y**], to map each row onto the corresponding coordinates in the unit-sum simplex, by the closure operator defined in terms of the matrix elements *x_ij_* of **X** by

$$x_{ij}^{\prime }={\left(C[X]\right)}_{ij}=\frac{x_{ij}}{\sum_{k=1}^{P}x_{ik}}$$

and similarly for **Y.** We here imputed nonzero values to replace zeroes using the multiplicative approach previously described (33), setting the δ imputed values to a single constant equal to the smallest nonzero element encountered across all of **X**′ and **Y**′. That is, given δ, we replaced **X**′ with **X**″ defined as

$$x_{ij}^{\prime \prime }=\{\begin{array}{cc} \delta , & |x_{ij}^{\prime }=0\\ (1-\sum_{k|x_{ik}=0}\delta )x_{ij}^{\prime } & |x_{ij}^{\prime }>0 \end{array}$$

and similarly replaced **Y**′ with **Y**″ in terms of the elements **Y**′. We found this approach to be more stable across various δ imputed values and more robust than adding pseudo counts. Given sequencing instrument limitations, this approach treats all zero counts as rounded zeroes where each taxon is assumed to be present but below detection level.

To analyze the overall gut microbiome compositional change in response to treatment, we quantified the relative change from the pre-exposure (**X**″∈ℝ^n×P^) to the post-exposure (**Y**″∈ℝ^n×P^) gut microbiome profile by the compositional perturbation operation **Z**″ = **Y**″ ⊖ **X**″ = C[**Y**″**/X**″] where the input to the closure operator C[] is the array of element-wise division of **Y**″ by the corresponding elements of **X**″ (34). To robustly handle compositional constraints on the perturbations encoded in **Z**″, simplify interpretation, and to avoid using a stepwise procedure on the high dimensional set of (P2)=26,335 pairwise log-ratios (PLR), an additive log-ratio (ALR) transformation was then applied to each sample (34): **X**‴ = ALR[**X**″], **Y**‴∈ℝ^n×(P-1)^ and **Z**‴ = ALR[**Z**″], **Z**‴∈ℝ^n×(P-1)^, defined in terms of selected reference taxa *x_ij_*″ or *z_ip_* by

$$x_{ij}^{\prime \prime \prime }={\left[ALR(X^{\prime \prime })\right]}_{ij}=\ln(\frac{x_{ij}^{\prime \prime }}{x_{ip}^{\prime \prime }}),j\neq p$$

$$z_{ij}^{\prime \prime \prime }={\left[ALR(Z^{\prime \prime })\right]}_{ij}=\text{ln}(\frac{z_{ij}^{\prime \prime }}{z_{ip}^{\prime \prime }}),j\neq p$$

and with the j = p column removed. To ensure the lower dimensional sets of ALRs (*P* – 1 = 229) adequately preserved the distance between samples using all PLR, the reference denominators *x_ip_*″ or *z_ip_*″ were selected such that the Procrustes matching to the higher-dimensional all-PLR configuration was maximized (35). Results from the selection of the top ALR denominator for the Procrustes correlation between the full PLR inter-sample distances for the pre-exposure and perturbation compositions are shown in **Figure S1**. To reduce computational complexity, the Aitchison distance was computed with a weighted centered log-ratio transformation in lieu of pairwise distances using all PLRs (36, 37). Procrustes analysis and Permutational multivariate ANOVA (PERMANOVA) were implemented using the vegan package in R (38). To identify a minimal taxa log-ratio signature, feature selection using machine learning was applied separately to **X**‴ and to **Z**‴, where the target outcome of interest was either strain or strain-food protein respectively (see *Machine Learning Analysis*). This resulted in the pre-exposure log-ratio $\hat{X}$ signature and perturbation log-ratio signature $\hat{Z}$. The pairwise distance matrix **A*_pert_*** between samples in $\hat{Z}$ were then calculated as

$$A_{pert}\sum_{k}{\left({\hat{z}}_{ik}-{\hat{z}}_{jk}\right)}^{2}$$

and **A*_presen_*** was similarly defined in terms $\hat{X}$ of Principal coordinate analysis using **A** was employed to visualize the composition of the pre-exposure or compositional perturbation gut microbiome between samples. PERMANOVA (39) was applied using **A** to compare: (1) the pre-exposure composition between strains (CC027/GeniUnc, C3H/HeJ), and (2) the compositional perturbation between strain-food protein groups (g = 10). Next, the vegan implementation of the PERMDISP2 procedure was applied to assess heterogeneity of variance between groups (39) and to confirm centroid and/or dispersion differences. Finally, pairwise multiple comparisons (compositional perturbation) using PERMANOVA with Benjamini-Hochberg (BH) multiple testing corrections were applied to confirm significant differences and to control the false discovery rate (FDR). The FDR was estimated using α = 0.06. Finally, the mean taxa log-ratio signature $\bar{S}\in {\mathbb{R}}^{g\times d}$ where *d* < *P* for each strain-food protein group in $\hat{Z}$ was computed.

Cluster analysis was performed on the strain-food protein groups with the Leiden algorithm (40) for community detection on graphs, and applied to a strain-food protein similarity graph *G* to model the similarity between samples within each strain-food protein group. To do so, *G* = (*V*, *E*, *W*) where *V* represents the set of strain-food protein groups, *E* represents the edge set formed by non-significant Pseudo-F values (from pairwise PERMANOVAs using BH corrected values, thereby indicating some possibility of similarity) with edge weights *W* given by corresponding pairwise Pseudo-F value between strain-food protein groups. To identify a biologically relevant set of clusters we tuned the resolution parameter used in the algorithm to satisfy two objectives: (1) minimize entropy between numbers of mice experiencing anaphylaxis (reactors) within each cluster and (2) maximize the number of clusters returned (which is achieved in the present case by maximizing the resolution parameter). With clusters defined in this way, the taxa log-ratios in were compared using the Kruskal-Wallis rank sum test to determine differences between clusters. Kruskall-Wallis p-values were adjusted for multiple comparison using BH (**Supplemental Table 1**).

#### Machine Learning Analysis

To reduce the number of features, we used a machine learning approach to select a reduced number of features while simultaneously assessing both the performance [repeated cross-validation (CV)] of the model and statistical significance (permutation testing) of the association between labels and features. To do this, all machine learning models used were trained with the random forest algorithm as implemented by the randomForest and caret packages in R (41, 42). Feature selection was cross-validated within the training folds and was done using the Boruta (43) algorithm in R (pre-exposure microbiome profile; binary outcome), or an implementation of the random forest recursive feature elimination (perturbation microbiome profile; mutli-classification). Model performance was computed using area under the receiver operating characteristic curve (AUROC) for both binary and multi-class classification metrics and was implemented using the pROC package in R (44). Performance was estimated using stratified 10 × 10-fold CV for binary classification and stratified 10 × 5-fold CV for mutli-classification. Given the small sample size and to ensure models were finding true associations between the labels and features, we performed additional permutation testing using the previously described procedure (45): we trained 100 additional models with permutated labels for each test and the empirical p-value was calculated as previously described as definition 1 (45). Feature importance metrics were calculated using the permutational accuracy metric during the final model training process. All log-ratio signatures were calculated using the full dataset after CV and permutation testing by first performing feature selection and then training the final model. All analyses were performed in R software 4.0.0 and are publicly available at https://github.com/andrew84830813/food_antigen_sensitization-mircobiome-genetically_susceptible_mice.git.

## Results

### CC027/GeniUnc Mice Become Sensitized to Peanut, Walnut, and Egg, but Not Milk, in the Absence of a Th2-Skewing Adjuvant

**Figure 1:**
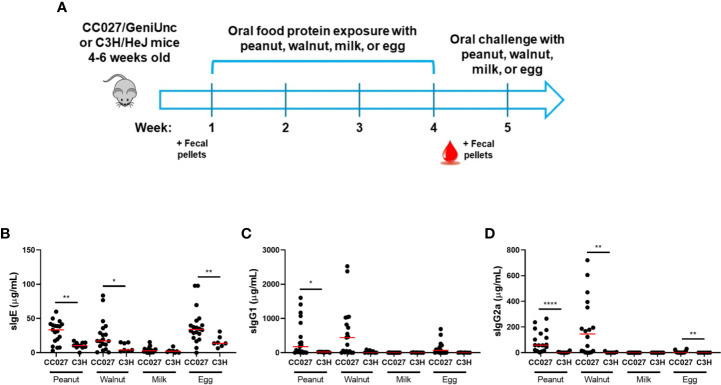
Antibody responses in CC027/GeniUnc and C3H/HeJ mice following oral exposure to peanut, walnut, milk, or egg. **(A)** Experimental design. **(B–D)**. Serum antigen-specific IgE **(B)**, IgG1 **(C)** and IgG2a **(D)** production in CC027/GeniUnc (n = 16–20 per food) and C3H/HeJ (n = 6–10 per food) mice at week 5. *p < 0.05, **p < 0.01, ****p < 0.0001, Mann Whitney U test, individual data are shown with a red line representing the median.

CC027/GeniUnc and C3H/HeJ mice differentially respond to oral peanut exposure (16). Here, we aimed to determine how these two strains of mice would react to various food proteins in the absence of a Th2-skewing adjuvant. To do this, mice were orally gavaged with peanut, walnut, milk, or egg protein once weekly for 4 weeks, and serum was collected after the exposure period to measure immunoglobulin production (**Figure 1A**). CC027/GeniUnc mice orally exposed to peanut, walnut, or egg produced high levels of antigen-specific IgE, IgG1, and IgG2a, indicating sensitization, while CC027/GeniUnc mice exposed to milk did not mount an IgE response. C3H/HeJ mice produced significantly lower quantities of serum peanut-, walnut-, and egg-specific IgE compared to CC027/GeniUnc, and near-undetectable milk-specific IgE (**Figures 1B–D**). Thus, CC027/GeniUnc mice were highly sensitized to peanut, walnut and egg, whereas C3H/HeJ mice were not.

### CC027/GeniUnc Mice Have Significantly Lower Total Fecal IgA Quantities and a Distinct Gut Microbiome Compared to C3H/HeJ Mice

**Figure 2:**
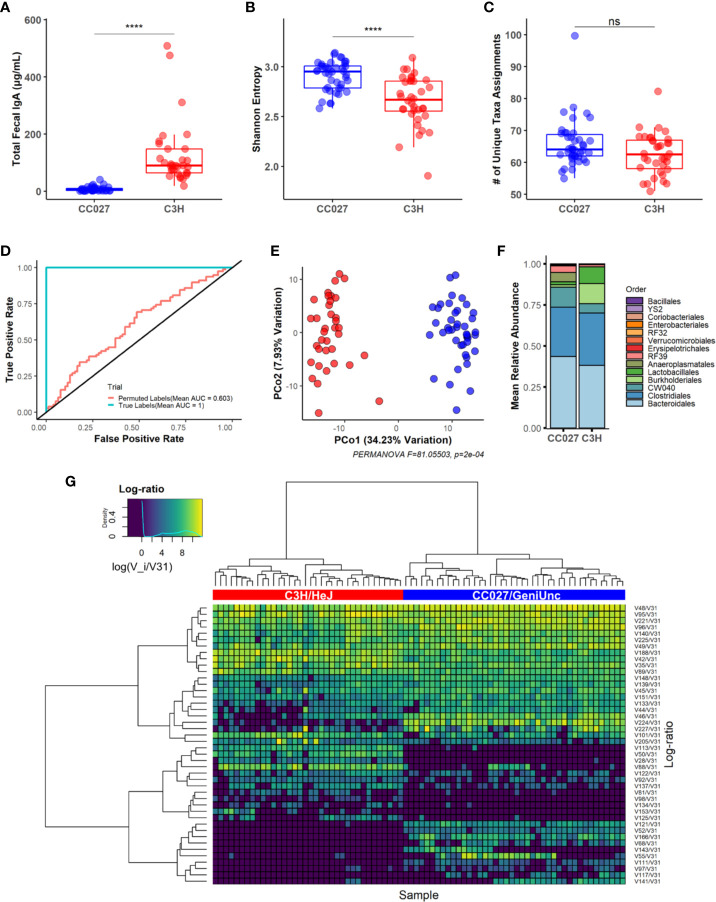
Fecal IgA and gut microbiome of naive CC027/GeniUnc and C3H/HeJ mice. **(A)** Total fecal IgA (CC027/GeniUnc: n = 39; C3H/HeJ: n = 32). **(B)** Shannon diversity between strains (CC027/GeniUnc: n = 42; C3H/HeJ: n = 36; used for all naive microbiome analysis). **(C)** Number of unique taxa. **(D)** Pooled Receiver Operating Characteristic (ROC) curve showing model performance with feature selection under true and permuted labels (strain). **(E)** Principal coordinate analysis plot of gut microbiome between CC027/GeniUnc (blue) and C3H/HeJ (red) mice. **(F)** Relative abundances based on order. **(G)** Heatmap showing between-strain log-ratio signature clustered with ward.D2 with Euclidean dissimilarity (CC027/GeniUnc: blue, C3H/HeJ: red; x-axis). **(A**–**C)** Wilcoxon rank-sum test, individual data are shown within a box plot showing median with IQ range. ****p < 0.0001.

Sensitization through the GI tract can be influenced by luminal IgA and the consortia of microbiota in the gut (10, 11, 13). Fecal pellets were collected from mice before the 4-week food protein exposure period to compare fecal IgA quantities and gut microbiome composition in naïve mice. CC027/GeniUnc mice had significantly lower total fecal IgA quantities compared to C3H/HeJ mice (**Figure 2A**). The vast majority of CC027/GeniUnc mice (34 of 39 mice) had <15 ng/ml of IgA, while IgA quantities in C3H/HeJ mice average 127 ng/ml (range: 18–509 ng/ml).

Increased gut microbiome diversity was observed in CC027/GeniUnc mice, as demonstrated by the Shannon index (**Figure 2B**). Notably, both strains had similar numbers of unique assigned taxa (**Figure 2C**, a complete list of taxa is in **Supplemental Table 2**). Compositional data analysis revealed the gut microbiome composition was significantly different between strains at baseline, using a combination of machine learning and beta diversity analysis (**Figures 2D, E**). Indeed, our machine learning model with feature selection was able to perfectly classify strains (AUROC = 1, 10 times repeated 10-fold CV, see *Materials and Methods*) when trained on the baseline gut microbiome composition. Marked visual differences in the average gut microbiome composition of CC027/GeniUnc and C3H/HeJ mice at the order level are shown in **Figure 2F**. Since there were clear gut microbiome differences between strains, we next sought to identify the specific taxa responsible for these strain differences. While our model was able to easily classify strains, we used permutation testing to ensure the model learned true associations between microbiome compositions and strain (**Figure S2**, see *Machine Learning Analysis*). To identify a strain-specific gut microbiome signature, we applied our cross-validated feature selection process using all samples. Indeed, we found log-ratios formed between the abundance of 44 taxa relative to a reference taxa, *Atopobium vaginae* (V31, used as the denominator in log-ratios, see *Compositional Data Analysis* for selection), to be important for explaining the difference between strains (**Figure 2G**). Specifically, random forest feature importance derived from the final model trained on the strain-specific signature revealed log-ratios of taxa from the *Anaeroplasma* (V224) genus and *Lachnospiraceae* (V113) family relative to *A. vaginae* were the top 2 among 44 features most important for classifying strain differences (**Figure S3**). In particular, univariate statistical analysis confirmed taxa from the *Anaeroplasma* genus were significantly more enriched relative to *A. vaginae* in CC027/GeniUnc mice than C3H/HeJ mice (all pairwise comparisons in **Figure S4A**). Conversely, univariate statistical analysis confirmed taxa from the *Lachnospiraceae* family were significantly more enriched relative to *A. vaginae* in C3H/HeJ mice rather than CC027/GeniUnc mice (**Figure S4B**). Together, these results indicate that differences in IgA levels between these two strains, as well as the composition and abundances of a subset of the gut microbiota distinguish each strain’s relative susceptibilities to sensitization.

### A Higher Number of T Follicular Helper and CXCR5^+^ B Cells in CC027/GeniUnc Mice Are Consistent With Higher Immunoglobulin Production Compared to C3H/HeJ

**Figure 3:**
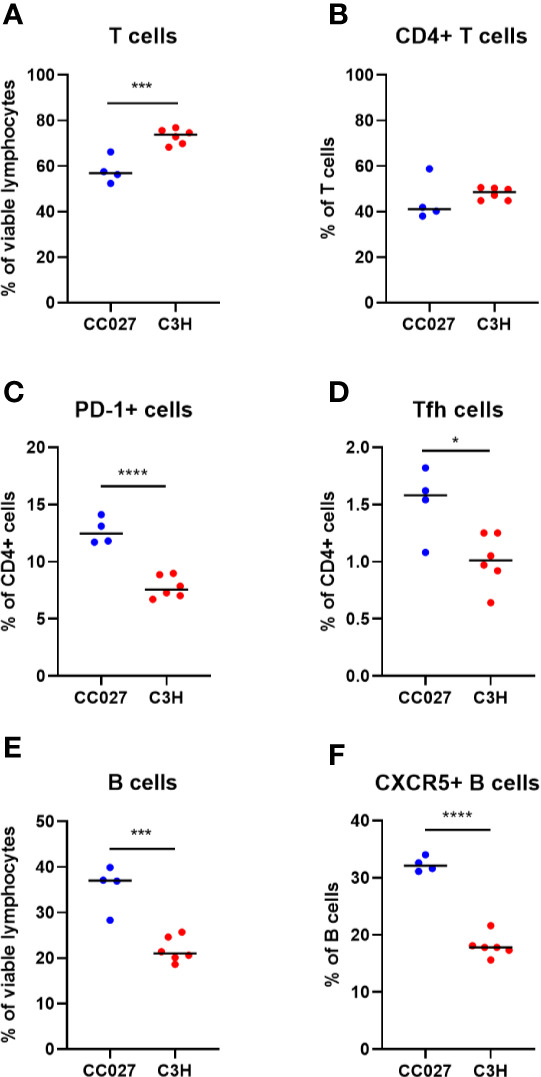
Lymphocyte subsets in mesenteric lymph nodes from CC027/GeniUnc (n = 4) and C3H/HeJ mice (n = 6). **(A)** T cells, **(B)** CD4+ T cells, **(C)** PD-1+ CD4+ cells, **(D)** T follicular helper cells, **(E)** B cells, and **(F)** CXCR5+ B cells. *p < 0.05, ***p < 0.001, ****p < 0.0001, Unpaired t test, individual data are shown with a black line representing the median.

Because the immunologic process of oral tolerance occurs in MLNs, we investigated T and B cells in the MLNs. Specifically, Tfh cells are integral in driving IgE production (46, 47), and their presence may help explain why CC027/GeniUnc mice become sensitized to peanut, walnut and egg. Following food protein oral exposure and challenge, MLNs were collected from mice for T and B cell analysis by flow cytometry (gating shown in **Figure S5**). CC027/GeniUnc mice had lower frequency of T cells overall, but similar frequency of CD4^+^ T cells compared to C3H/HeJ mice (**Figures 3A, B**). CC027/GeniUnc mice also had higher frequency of antigen-experienced, PD-1^+^CD4^+^ T cells (**Figure 3C**). Importantly, the frequency of Tfh cells was higher in CC027/GeniUnc mice compared to C3H/HeJ mice (**Figure 3D**). CC027/GeniUnc mice also had higher frequency of B cells and CXCR5^+^ B cells (**Figures 3E, F**), suggesting that the number of germinal center B cells is higher, resulting in higher immunoglobulin production in CC027/GeniUnc mice. Overall, the increased abundances of Tfh cells and CXCR5^+^ B cells may lead to higher IgE production in CC027/GeniUnc mice.

### CC027/GeniUnc Mice Experience Anaphylaxis Upon Oral Challenge to Peanut and Walnut, but Not Milk or Egg

**Figure 4:**
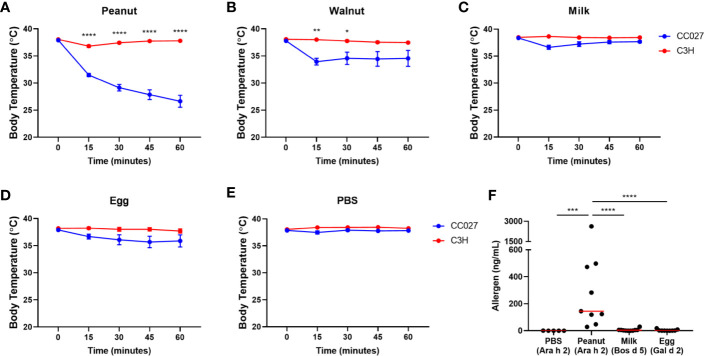
Oral challenge results. **(A–E)** Body temperature changes in CC027/GeniUnc (blue, n = 10 per food) and C3H/HeJ (red, n = 7–9 per food) mice challenged to peanut **(A)**, walnut **(B)**, milk **(C)**, egg **(D)**, and PBS **(E)**. **(F)** Serum allergen quantities post-challenge. Allergens in parentheses represent the allergen quantified for each group. *p < 0.05, **p < 0.01, ***p < 0.001, ****p < 0.0001, two-way ANOVA, data is represented by the mean ± SEM for each time point. Statistics are shown for groups that reacted upon challenge (>3°C decrease). For allergen quantities: Mann Whitney U test, individual data are shown with a red line representing the median.

While IgE production indicates that CC027/GeniUnc mice were sensitized to peanut, walnut and egg, oral food challenges are required to confirm an allergy to these foods. Mice were challenged to either peanut, walnut, milk or egg proteins *via* oral gavage following the exposure period. CC027/GeniUnc mice experienced severe anaphylaxis (>5°C decrease) to peanut (**Figure 4A**), and mild anaphylaxis (>3°C decrease) to walnut (**Figure 4B**), but did not experience allergic reactions (<3°C decrease) to milk, egg or PBS (**Figures 4C–E**). C3H/HeJ mice did not experience reactions to any oral challenge. Although CC027/GeniUnc mice had increased IgE production in response to peanut, walnut and egg, mice only experienced anaphylaxis to peanut and walnut, suggesting that there are additional factors besides IgE production that contribute to reactivity.

We previously reported that CC027/GeniUnc mice sensitized with peanut and cholera toxin have detectable peanut allergen, Ara h 2, in their serum following oral peanut challenge (16), suggesting increased antigen absorption may contribute to anaphylaxis to peanut. To determine whether cholera toxin-free exposure to each food led to antigen absorption post-challenge, we quantified major serum allergens in peanut (Ara h 2), milk (Bos d 5), and egg (Gal d 2) *via* ELISA in peanut-, milk-, and egg-exposed mice, respectively (**Figure 4F**). Consistent with our previous results, CC027/GeniUnc mice sensitized to peanut had detectable levels of Ara h 2 in serum following oral peanut challenge. Contrarily, Bos d 5 and Gal d 2 were not detected in mice following milk and egg challenge, respectively. Validated ELISA reagents were not available for any major walnut allergen, and allergens were not quantified in walnut-sensitized mice. Together these results indicate that CC027/GeniUnc mice have increased antigen absorption in the gut following peanut, but not milk or egg, challenge, which is likely an important factor leading to anaphylaxis.

### Fecal Immunoglobulin Quantities in CC027/GeniUnc Mice Are Associated With Anaphylaxis Upon Oral Challenge

**Figure 5:**
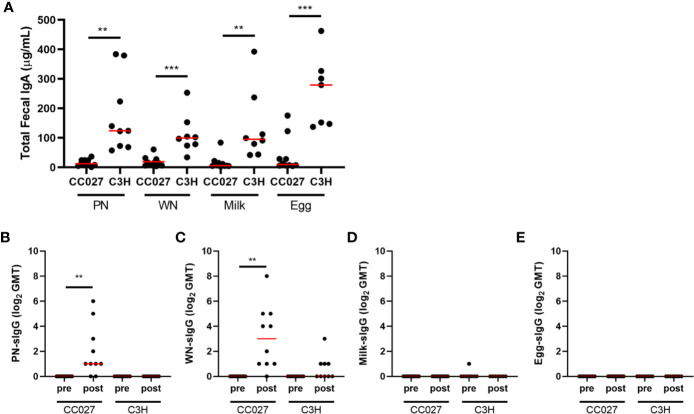
Fecal immunoglobulin quantities. **(A)** Total fecal IgA measured in peanut-, walnut-, milk-, or egg-exposed CC027/GeniUnc (n = 10 per food) and C3H/HeJ (n = 7–9 per food) mice. **(B–E)** Fecal peanut- **(B)**, walnut- **(C)**, milk- **(D)**, and egg- **(E)** specific IgG (represented as log_2_ of the geometric mean titer (GMT)) in food protein-exposed CC027/GeniUnc (n = 9–10 per food) or C3H/HeJ (n = 7–9 per food) mice. **p < 0.01, ***p < 0.001, Unpaired t test (IgA) and Wilcoxon test (IgG), individual data are shown with a red line representing the median.

To further investigate potential gut absorption differences between groups, fecal pellets collected after the food protein exposure period were analyzed for total fecal IgA. CC027/GeniUnc mice have very low total fecal IgA, while C3H/HeJ mice have higher total fecal IgA regardless of antigen exposure (**Figure 5A**). Antigen-specific IgA was not detected, regardless of strain or food protein exposure. Antigen-specific IgG was also measured in fecal pellets. Interestingly, CC027/GeniUnc mice sensitized to peanut or walnut had increased fecal antigen-specific IgG following exposure, while CC027/GeniUnc mice exposed to milk and egg had no detectable antigen-specific fecal IgG (**Figures 5B–E**). These results associated with the CC027/GeniUnc anaphylaxis outcomes and may suggest increased antibody production in the gut or the translocation of antibody across the gut barrier to the lumen.

### Microbial Compositions Differ Among CC027/GeniUnc Mice Exposed to Peanut, Walnut, Milk, or Egg

**Figure 6:**
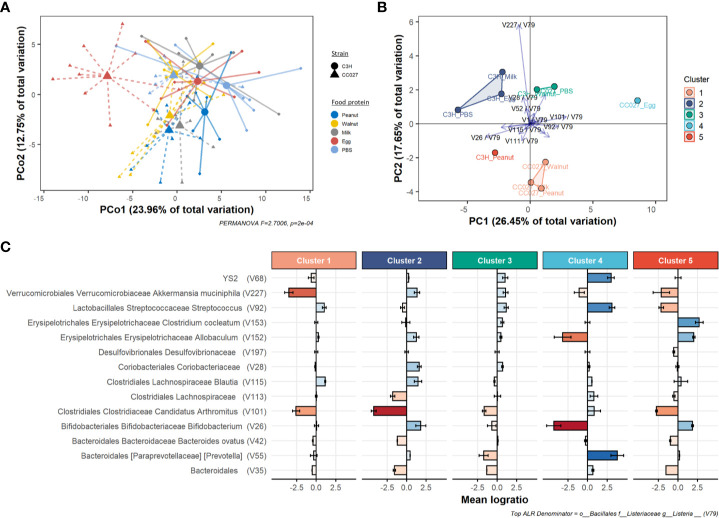
Gut microbiome changes after food protein exposure in CC027/GeniUnc (n = 7–10 per food) and C3H/HeJ (n = 7–8 per food) mice. **(A)** PCoA plot of compositional perturbation explaining 36.71% of total variation grouped by strain-food protein. **(B)** PCA biplot showing relative location of clusters and how the mean strain-food protein log-ratios within the perturbation signature contribute to differences between groups. **(C)** Depletion or enrichment of specific taxa relative to *Listeria* within each cluster. Error bars reflect 95% confidence intervals of the mean log-ratio value for all samples within each cluster. All taxa information is included in the following sequence: order, family, genus, species.

Given that gut microbial composition is affected by dietary antigens, we hypothesized that enteral exposure to different foods in CC027/GeniUnc mice would lead to distinct changes of microbial compositions. To compare the effects of different food proteins, the relative changes in gut microbiome before and after exposure (i.e., perturbation, see *Materials and Methods*) were further analyzed. Accordingly, a multi-classification model with feature selection was trained on the microbiome perturbation composition in response to food protein exposure, and a statistically significant association (AUROC = 0.84, p = 0.009, see *Materials and Methods*, **Figure S6**) was identified between the strain-food protein interaction (i.e., CC027-peanut, C3H-peanut, etc.) and the microbiome perturbation. Using this feature selection process on all samples we identified a reduced signature of 23 taxa log-ratios representative of how each strain’s gut microbiome responded to the food exposure. A Principal Coordinate Analysis (PCoA) plot using the log-ratio signature shows perturbation dissimilarities based on food exposure and strain (**Figure 6A**). PERMANOVA analysis confirmed there were some significantly distinct gut microbiome responses to food exposure based on strain (Pseudo-F = 2.7006, p < 0.0001). Multivariate dispersions between strain-food protein interactions were analyzed with PERMDISP2 (**Figure S7**), and the results indicated differences in multivariate dispersion were not significant (Pseudo-F = 1.0100, p = 0.4409) and instead that there were only differences among the location of group centroids.

To compare perturbation responses, we used similarity graphs (see *Compositional Data Analysis*) to cluster each strain-food protein group. From this, we identified five clusters (**Figure 6B** and **Figure S8**) of responses to food protein exposure. Notably, our clusters resulted in a disproportionate number of anaphylactic mice (90%, n = 16) being concentrated in cluster 1 (**Figure S9A**). Further, permutation testing revealed that our five cluster assignments clustered anaphylactic mice significantly better than random clustering (p = 0.0146, **Figure S9B**). These results indicate our log-ratio signature not only distinguishes differences between strain-food protein perturbations but also may explain key changes in the gut microbiome associated with increased risk of anaphylaxis.

To explore the location-only effects, principal component analysis (PCA) of the mean log-ratio signature of each strain-food protein group revealed strong separation between clusters (**Figure S10**). PCA biplot analysis revealed key log-ratios responsible for cluster separation (**Figure 6B**) defined primarily by *Bifidobacterium* (V26), *Akkermansia* (V227), and *Candidatus Arthromitus* (V101) relative to *Listeria* (V79, see *Compositional Data Analysis* for selection). We next sought to understand how each cluster differed based on each log-ratio of the perturbation signature. Kruskal-Wallis testing revealed 13 log-ratios had some statistically significant difference in values across clusters (**Supplemental Table 3**). Additionally, random forest variable importance shows the relative importance of each log-ratio for associating strain-food protein to microbiome response (**Figure S11**). Specific taxa responsible for differences between groups are shown in **Figure 6C**, which demonstrates an average depletion or enrichment of taxa relative to *Listeria* (V79) in each group. These results indicate key taxa may be critical for distinguishing groups of mice that react upon oral food challenge.

## Discussion

Food allergies are thought to develop from oral tolerance failure, but the underlying mechanisms leading to sensitization are widely unknown. In mouse models, oral tolerance is typically broken by using mucosal adjuvants like cholera toxin or Staphylococcal enterotoxin B. However, we recently identified a genetically susceptible strain (CC027/GeniUnc) that does not require an adjuvant for peanut sensitization (16). Here, we focused on understanding the mucosal immunology in this CC027/GeniUnc strain that allows for enteric sensitization by analysing fecal IgA, antigen absorption, microbiome composition and MLN lymphocyte subsets after exposure to peanut, walnut, milk, or egg.

CC027/GeniUnc mice were orally sensitized to peanut, walnut and egg based on their immunoglobulin production, but only reacted upon oral challenge to peanut and walnut. Furthermore, CC027/GeniUnc mice had detectable serum Ara h 2 post-oral peanut challenge, while Bos d 5 and Gal d 2 were not detected in milk- or egg-challenged mice. Together, these results suggest that antigen absorption may play a role in reactivity in sensitized mice. Systemic absorption was previously shown to be required for anaphylaxis (48), which is consistent with our findings. Prior to absorption, antigens encounter enzymes and an acidic environment, which can lead to extensive hydrolysis of the antigen. Therefore, the biochemical properties of allergens are critical in maintaining the capacity to cross-link IgE. The major peanut allergen, Ara h 2, is a 2S albumin and is resistant to proteolysis, which likely contributes to its absorption and reaction susceptibility (49, 50). Similarly, the 2S albumins that are major allergens in tree nuts are also quite stable, which may explain walnut-induced reactions in this model.

With enteral exposure, antigen presentation occurs in gut-draining MLNs. These immunologic sites can lead to tolerance or allergy. Mounting evidence demonstrates that Tfh cells are the main source of IL-4 leading to IgE production (51). Since CC027/GeniUnc but not C3H/HeJ mice produced IgE against enteral antigens, we hypothesized that there may be more Tfh cells in MLNs. Indeed, CC027/GeniUnc mice had a higher frequency of Tfh cells compared to C3H/HeJ, despite having lower T cell frequencies. Furthermore, CC027/GeniUnc mice had more CD4^+^ T cells expressing PD-1, indicating more antigen presentation leading to antigen-experienced T cells occurs compared to C3H/HeJ mice. CC027/GeniUnc mice also had a higher frequency of MLN CXCR5^+^ B cells compared to C3H/HeJ. CXCR5 is critical for homing to lymph node follicles and is therefore important for class-switching and IgE production (52). These results suggest increased frequencies of MLN Tfh and CXCR5^+^ B cells in the CC027/GeniUnc mice may lead to higher IgE production compared to C3H/HeJ mice.

Mucosal IgA is thought to be protective against food allergies by preventing translocation of allergen through the gut barrier. In humans, selective IgA deficiency has been associated with allergic diseases, including food allergy (53, 54). Here, CC027/GeniUnc mice have very low levels of total fecal IgA compared to C3H/HeJ mice prior to food protein exposure, which may lead to less neutralization of allergen and more allergen absorption in the gut. Of note, fecal antigen-specific IgA was undetectable in either strain. Together, our results are consistent with a recent report demonstrating that cholera toxin was required for fecal antigen-specific IgA production, and that Tfh cells were not required for mucosal IgA production (11).

The main contributor to the gut mucosa is the microbiome, which has been linked to susceptibility to food allergy. We rigorously analyzed the gut microbiome with a combination of compositional data and machine learning approaches and found marked compositional differences between CC027/GeniUnc and C3H/HeJ mice at baseline. Furthermore, we found the gut microbiome of CC027/GeniUnc mice responded differently when exposed to peanut, walnut, milk, and egg when compared to C3H/HeJ mice. Importantly, separation of the five clusters in response to food protein exposure was driven primarily by specific changes in relative abundance of *Bifidobacterium*, *Akkermansia*, and *Candidatus Arthromitus* relative to *Listeria*. In our analysis, the peanut-, walnut- and milk-exposed CC027/GeniUnc mice (cluster 1 in **Figures 6B, C**), were defined by a depletion in *Akkermansia* bacteria relative to *Listeria*. *Akkermansia*, in particular, is known to play a critical role in gut barrier integrity through mucus layer production (55, 56); the relative depletion of *Akkermansia* is consistent with the presumed decreased gut barrier function in peanut- and walnut-sensitized CC027/GeniUnc mice. Moreover, given the clustering assignments, we found that cluster 1 (peanut-, walnut-, and milk-exposed CC027/GeniUnc mice) disproportionately contained ~90% of all mice that reacted upon challenge. Although milk-exposed CC027/GeniUnc mice did not demonstrate similar gut barrier permeability, they also did not produce any milk-specific immunoglobulins, which is a prerequisite component of allergy. Similar to cluster 1, peanut-exposed C3H/HeJ mice (cluster 5) had a notable decrease in *Akkermansia*, but also an increase in *Bifidobacterium*, which together with IgA production, may be protective against peanut allergy. The gut microbiome of egg-sensitized CC027/GeniUnc mice was characterized by the enrichment of taxa from the order YS2, and *Prevotella* and *Streptococcus* genera, and depletion of taxa from the *Bifidobacterium* and *Allobaculum* genera relative to *Listeria*. Since egg-sensitized CC027/GeniUnc mice did produce increased egg-specific IgE, perhaps the egg-induced microbiome changes did not further facilitate an *Akkermansia*-depleted, and therefore weaker, gut barrier that we believe is associated with the reactions in the peanut- and walnut-sensitized CC027/GeniUnc mice. Together these results suggest increased production of specific IgE, decreased total fecal IgA, and alterations of *Bifidobacterium* and *Akkermansia* communities in the gut are all associated with sensitization of food proteins and risk of anaphylaxis on exposure.

Due to the dynamic nature of the gut environment, multiple factors are known to contribute to sensitization susceptibility. The results presented here suggest an interactive role between food antigens and the local gut environment, including IgA and microbiome, leading to tolerance or sensitization. Specifically, CC027/GeniUnc mice have very low levels of total fecal IgA and a unique microbiome, which together may lead to increased antigen uptake. Then, in the MLNs, Tfh cells, which are also increased in CC027/GeniUnc mice, may drive the CXCR5^+^ B cells to class-switch into IgE-producing cells. Once animals are sensitized, increased gut permeability is a potential factor allowing for systemic absorption and anaphylaxis to occur. Based on the immunologic responses generated after food protein exposure in the absence of a Th2-skewing adjuvant, this model in CC027/GeniUnc mice provides a useful platform for future food allergy studies.

## Data Availability Statement

The datasets presented in this study can be found in online repositories. The names of the repository/repositories and accession number(s) can be found in the article/**Supplementary Material**.

## Ethics Statement

The animal study was reviewed and approved by Institutional Animal Care and Use Committee at the University of North Carolina at Chapel Hill.

## Author Contributions

JS, MK, KO, MF, HS, and AB conceived project and contributed to study design. JS, MK, BJ-W, MA-P, TM, RI, JK, and ES performed experiments and analyzed data. AH performed compositional and machine learning analysis in consultation with JS, MK, and PM. JS and MK wrote the manuscript. All authors contributed to the article and approved the submitted version.

## Funding

JS is funded by a T32 Allergy/Immunology Training Grant (AI007062) through Duke University and University of North Carolina (UNC) at Chapel Hill. Research was supported by UNC SOM Office of Research and TraCS Translational Team Science Award (grant nos. TTSA017P1 and TTSA017P2; to AB, MK, and MF), American Research Foundation for Nut Allergies (to JS and MK). ES is funded by T32 DK007737 (CGIBD T32) and Thurston Arthritis Research Center and NIAID Loan Repayment Program. AH is funded as a Howard Hughes Medical Institute Gilliam Fellow.

## Conflict of Interest

The authors declare that the research was conducted in the absence of any commercial or financial relationships that could be construed as a potential conflict of interest. 
