# Supplementary material for: Fecal IgA, Antigen Absorption, and Gut Microbiome Composition Are Associated With Food Antigen Sensitization in Genetically Susceptible Mice
Source: Front Immunol. 2021 Jan 19;11:599637. doi: 10.3389/fimmu.2020.599637 (PMC7850988; doi:10.3389/fimmu.2020.599637)
Supplement: Supplementary file 1 [file DataSheet_1.docx]

Supplementary Material

# Supplementary Data

All compositional and machine learning analyses were performed in R software 4.0.0 and are publicly available at https://github.com/andrew84830813/food_antigen_sensitization-mircobiome-genetically_susceptible_mice.git.

# Supplementary Figures and Tables

## Supplementary Figure Legends

**Supplementary Figure 1**. Top ALR denominator selection by Procrustes correlation maximization. A. Pre-exposure: Selection of top ALR denominator by Procrustes Correlation (top) PLR Inter-sample distances vs. top ALR denominator inter-sample distances B. Perturbation: Selection of top ALR denominator by Procrustes Correlation (top) PLR Inter-sample distances vs. top ALR denominator inter-sample distances.

**Supplementary Figure 2**. Significance of random forest multi-classification model trained on pre-exposure microbiome between strain. Permutation test with AUROC under null hypothesis of no association between strain (labels) and pre-exposure microbiome.

**Supplementary Figure 3**. Random forest permutational importance when trained on pre-exposure gut microbiome between strains. Enriched denotes statistically significant result from univariate Wilcoxon rank-sum test (when adjusted from multiple comparison using BH method) revealed value of log-ratio relative to V31 was higher in the respective strain.

**Supplementary Figure 4**. Univariate analysis of pre-exposure log-ratio signature by strain. Multiple comparisons between strains using Wilcoxon Rank-Sum Test with BH p-value correction.

**Supplementary Figure 5**. A. Gating strategy for T follicular helper (Tfh) cells. B. Shift in Tfh cell population using anti-CXCR5 compared to isotype control.

**Supplementary Figure 6**. Significance of random forest multi-classification model trained on compositional perturbation between strain-food protein. Permutation test with AUROC under null hypothesis of no association between strain-food protein (labels) and compositional perturbation.

**Supplementary Figure 7**. PERMDISP2 demonstrating heterogeneity of multivariate dispersion between strain-food protein groups.

**Supplementary Figure 8**. Clustering using similarity graph with Leiden algorithm. A. Cluster size as a function of resolution parameter. B. Minimization Entropy (number of anaphylactic mice within cluster) while maximizing resolution parameter. C. Similarity graph between strain-food protein groups.

**Supplementary Figure 9**. Clustering identifies similarity of gut microbiome perturbations among anaphylactic mice. A. Distribution of anaphylactic mice from Leiden clustering. B. Permutation test assessing significance of actual entropy under the null hypothesis that there is no association between entropy within clustered anaphylactic mice and clustering assignments.

**Supplementary Figure 10**. Heatmap of mean log-ratio signature of each strain-food protein group. Clusters correspond to groups in Figure 6B and 6C.

**Supplementary Figure 11**. Random forest permutational importance by group when trained on perturbation gut microbiome between strain-food protein groups.

## Supplementary Table Legends

**Supplementary Table 1**. Statistical comparison of perturbation between strain-food protein groups.

**Supplementary Table 2**. Complete list of taxa used for analysis.

**Supplementary Table 3**. Statistical comparison of taxa between clusters.
