# Supplementary figures and images for: Fecal IgA, Antigen Absorption, and Gut Microbiome Composition Are Associated With Food Antigen Sensitization in Genetically Susceptible Mice
Source: Front Immunol. 2021 Jan 19;11:599637. doi: 10.3389/fimmu.2020.599637 (PMC7850988; doi:10.3389/fimmu.2020.599637)

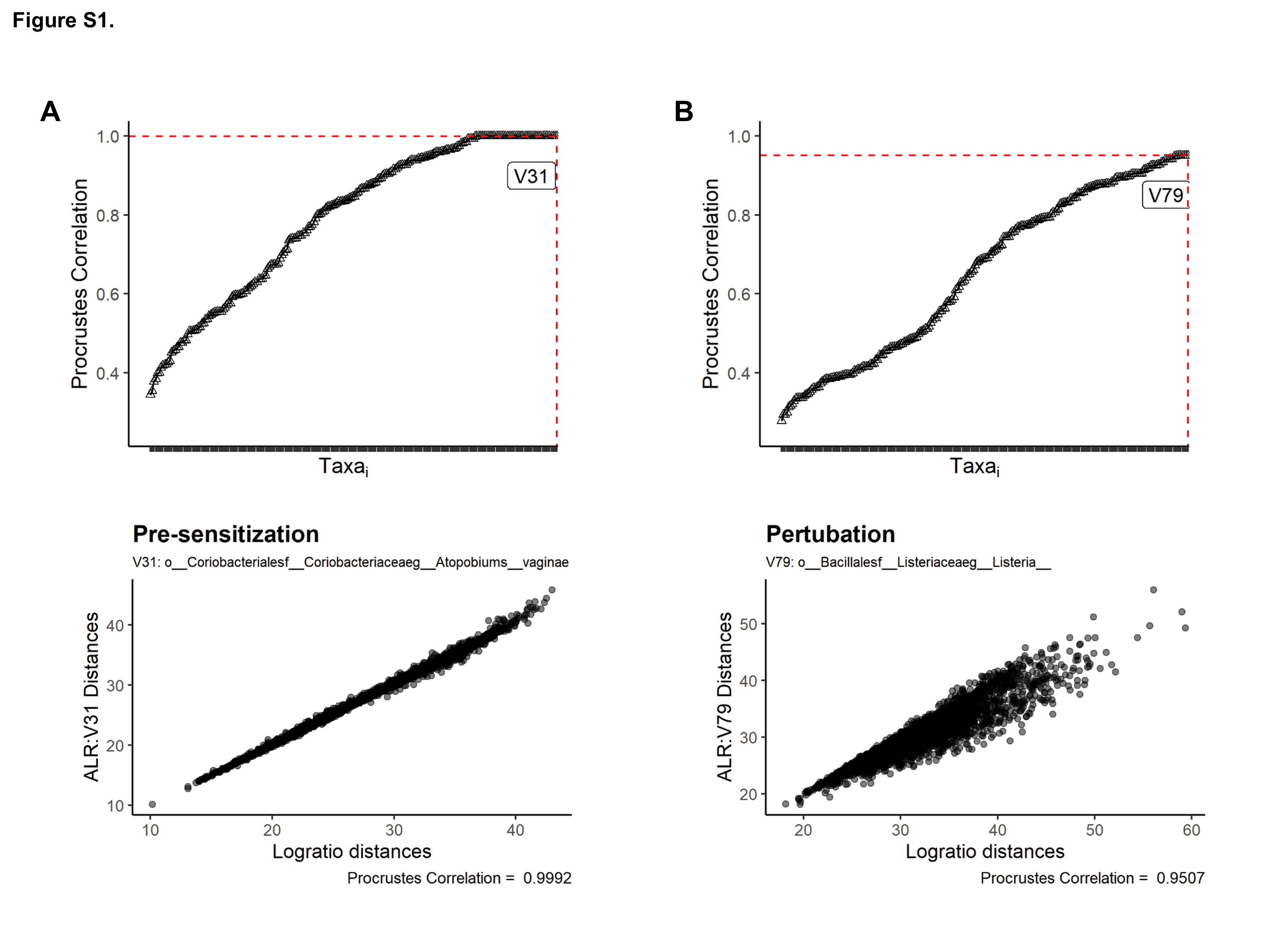

Supplement: Supplementary file 2 [file Image_1.tif]

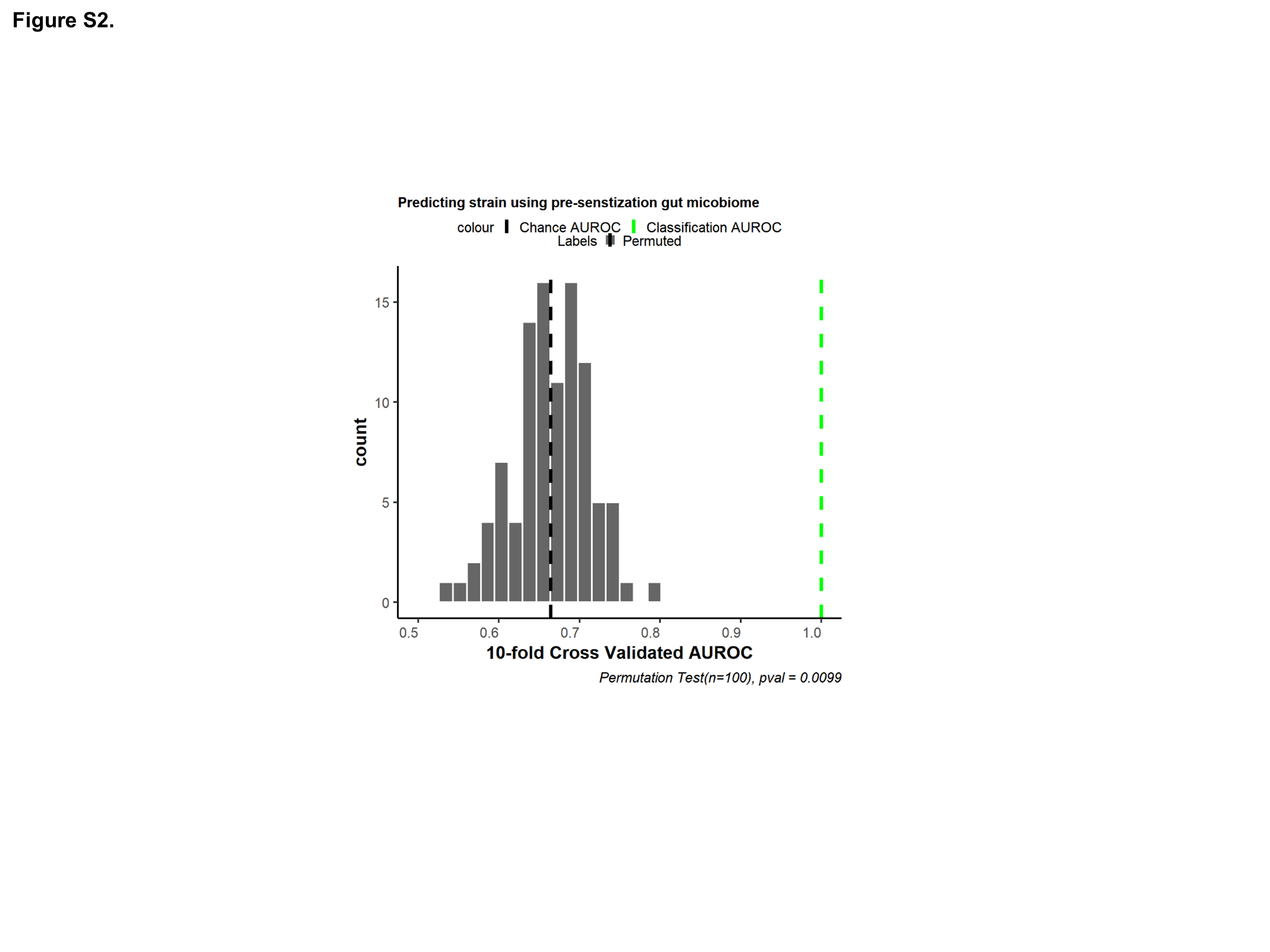

Supplement: Supplementary file 3 [file Image_2.tif]

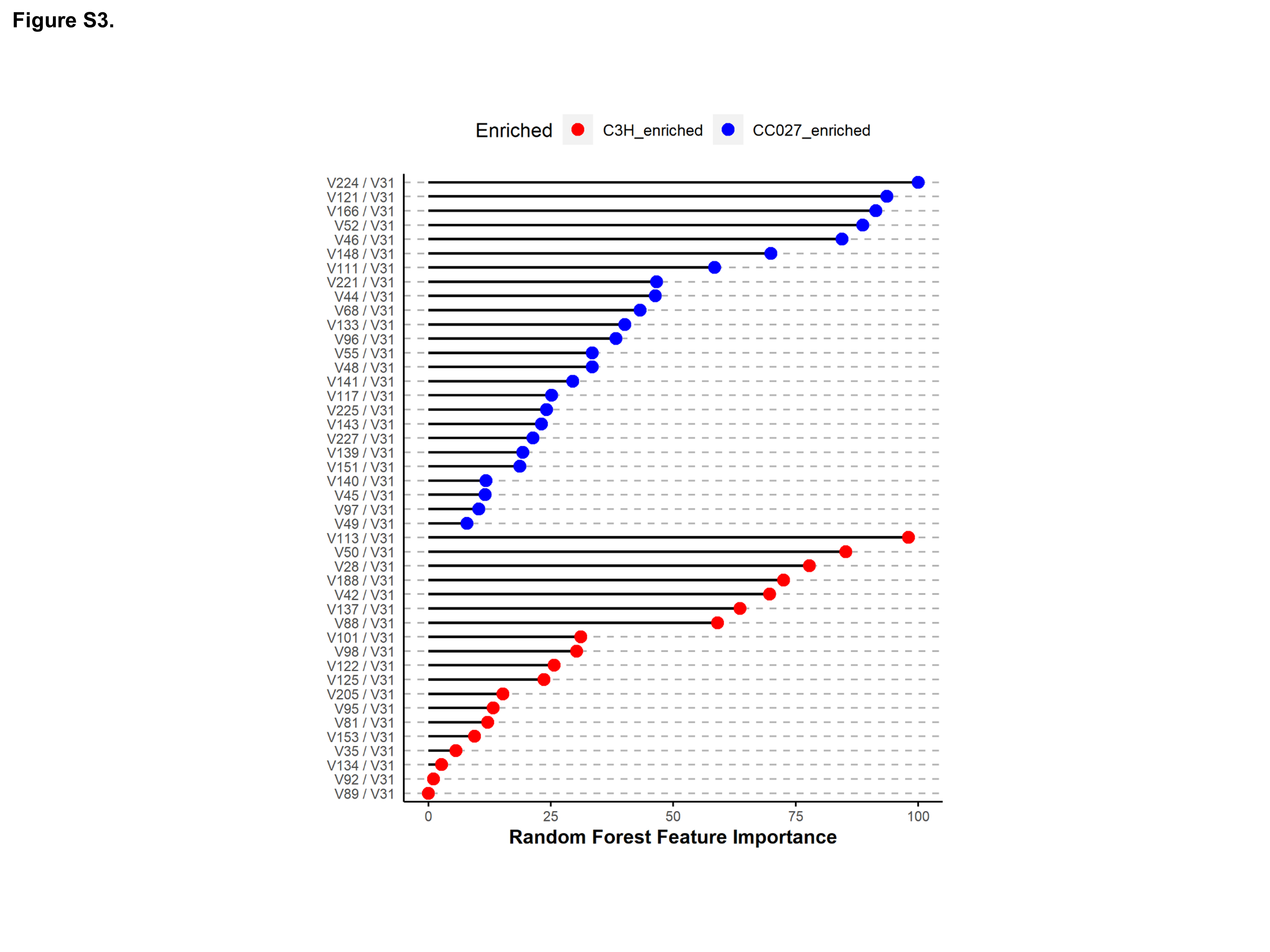

Supplement: Supplementary file 4 [file Image_3.tif]

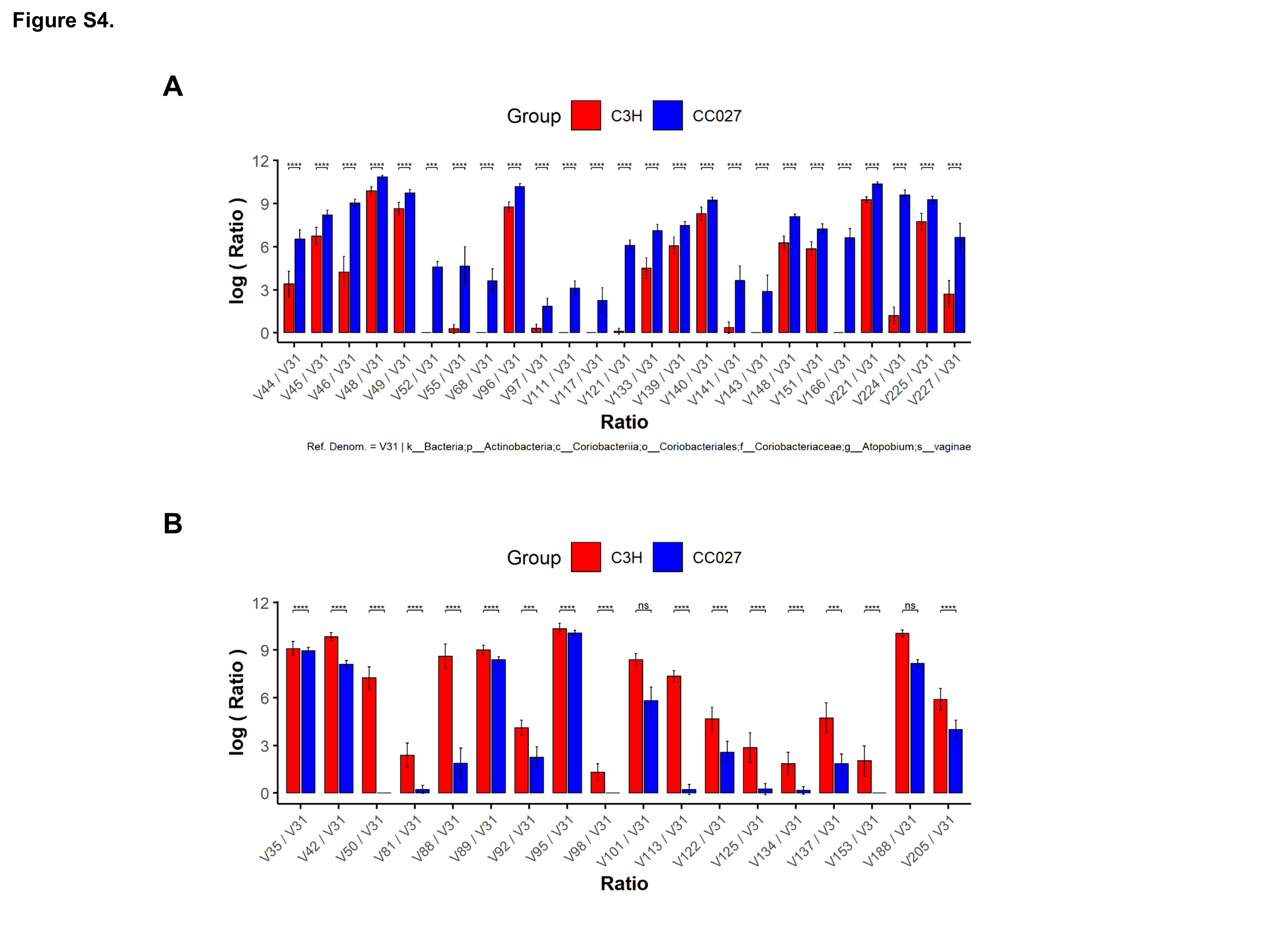

Supplement: Supplementary file 5 [file Image_4.tif]

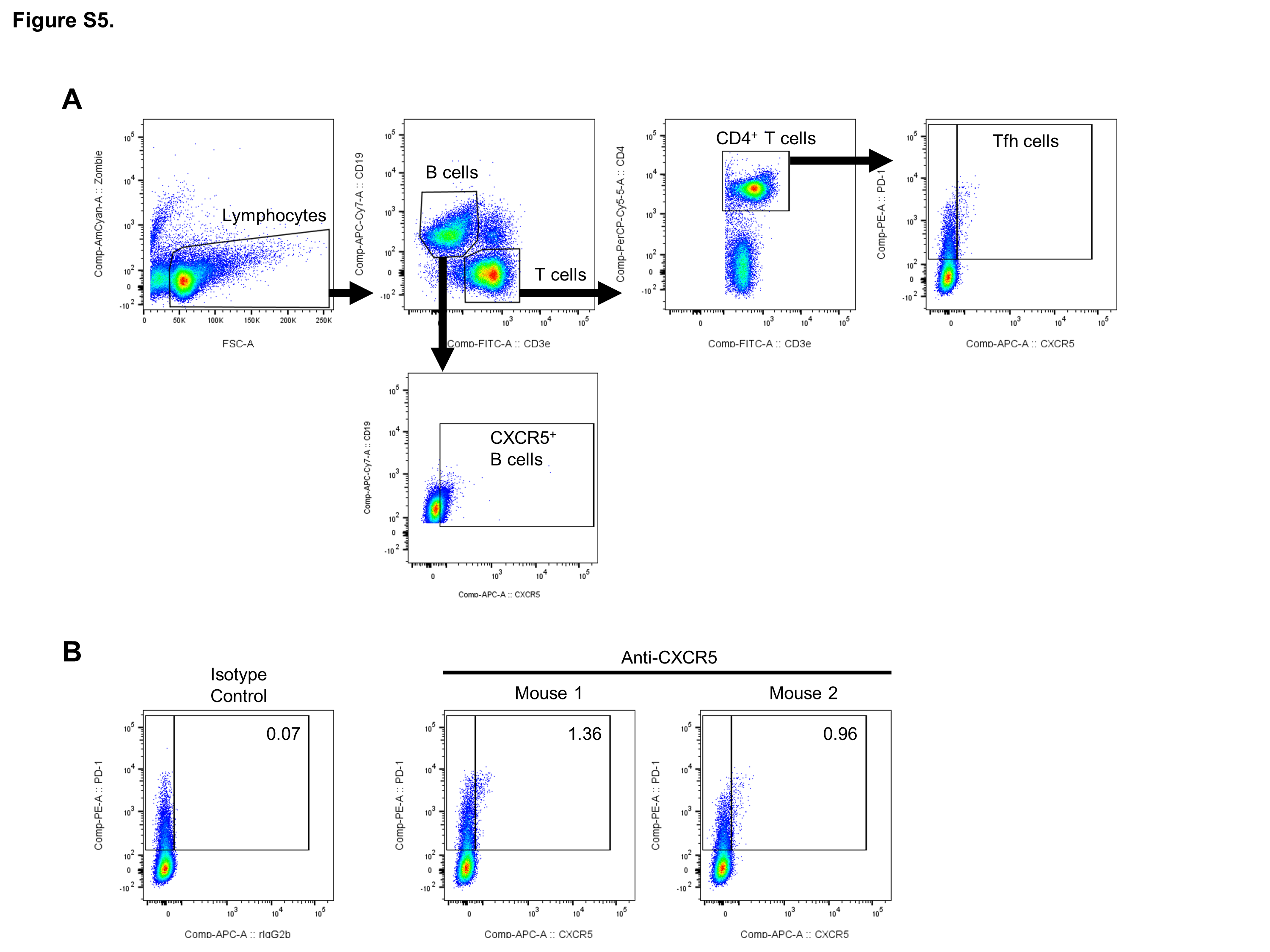

Supplement: Supplementary file 6 [file Image_5.tif]

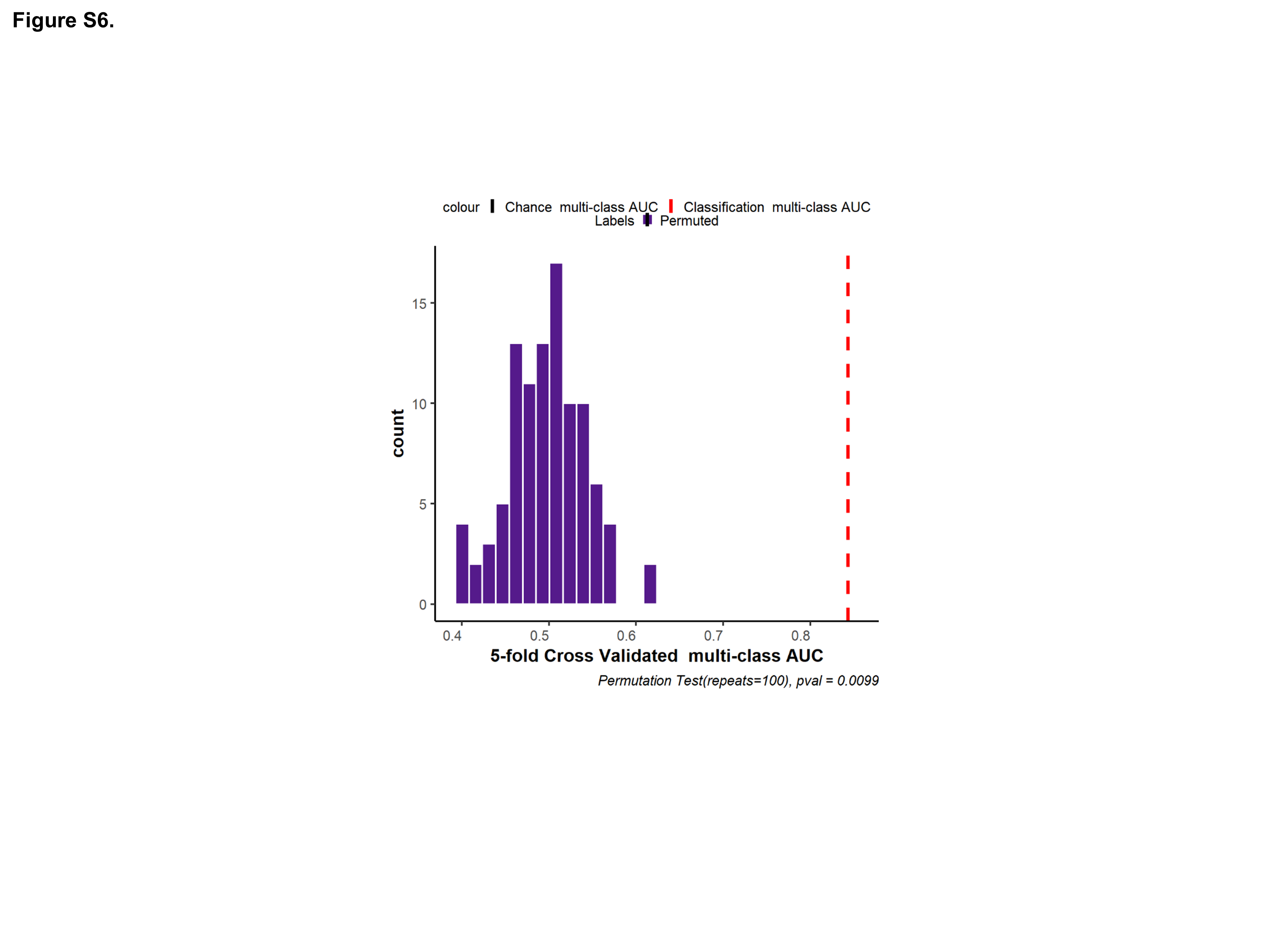

Supplement: Supplementary file 7 [file Image_6.tif]

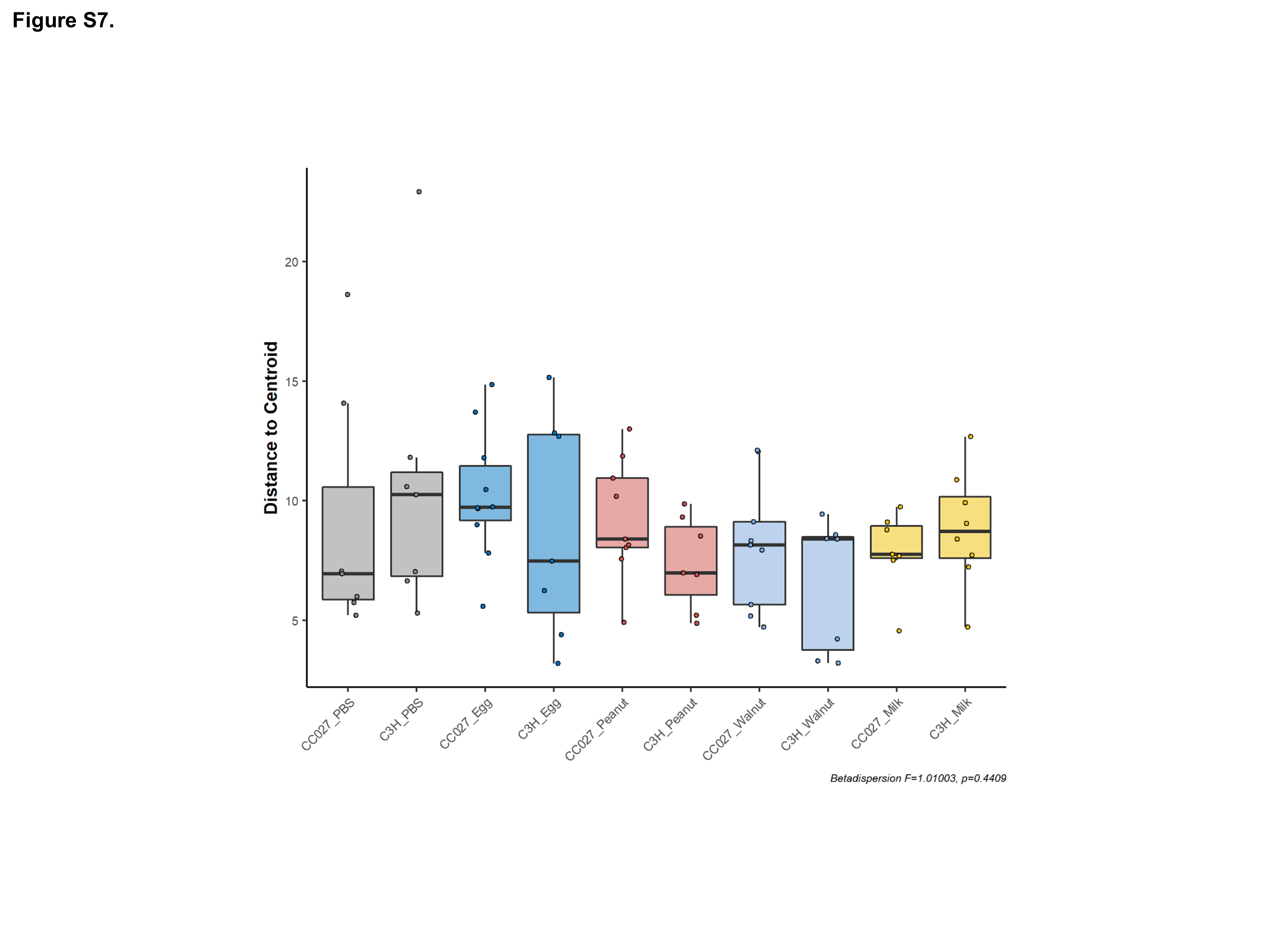

Supplement: Supplementary file 8 [file Image_7.tif]

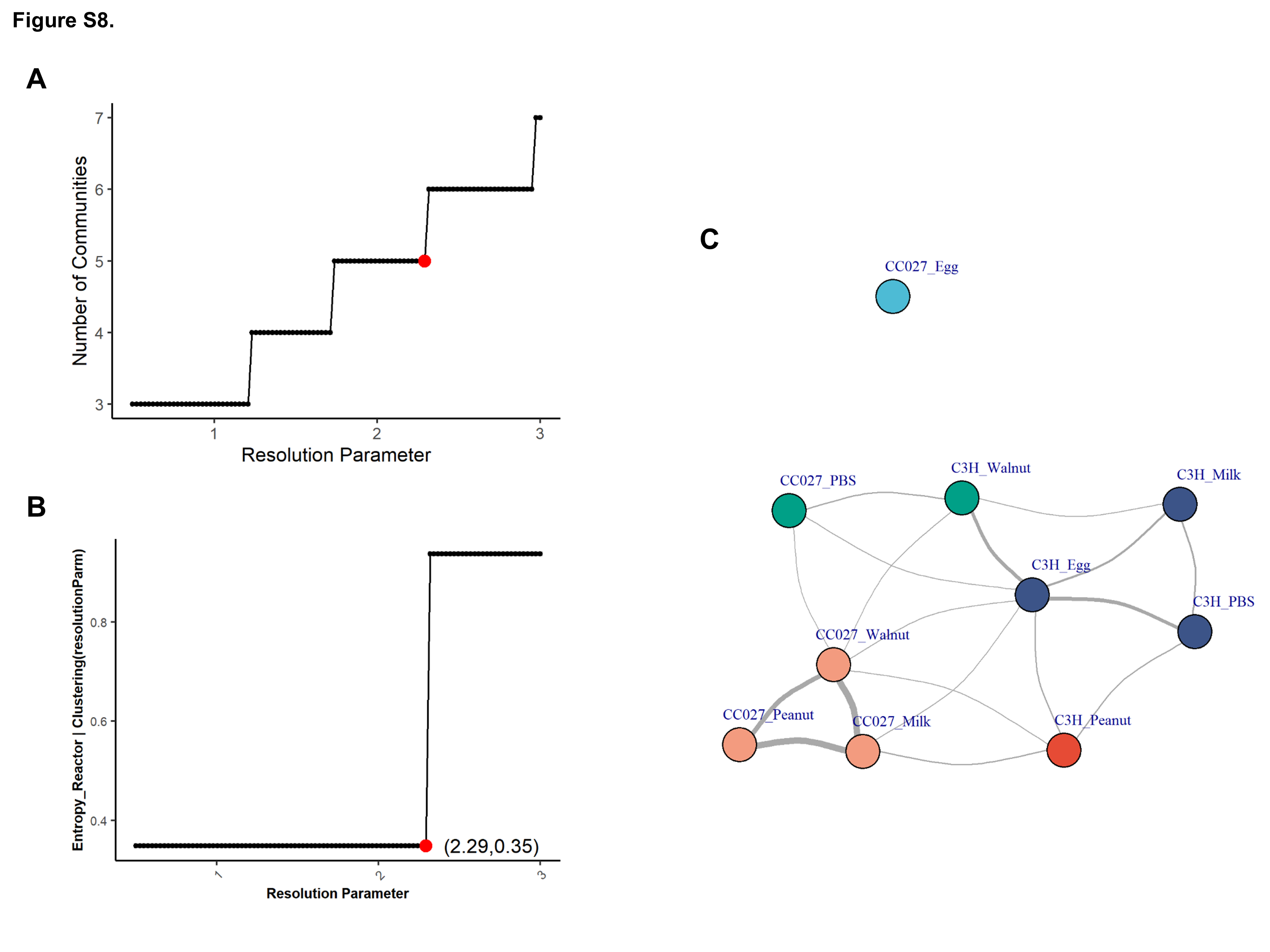

Supplement: Supplementary file 9 [file Image_8.tif]

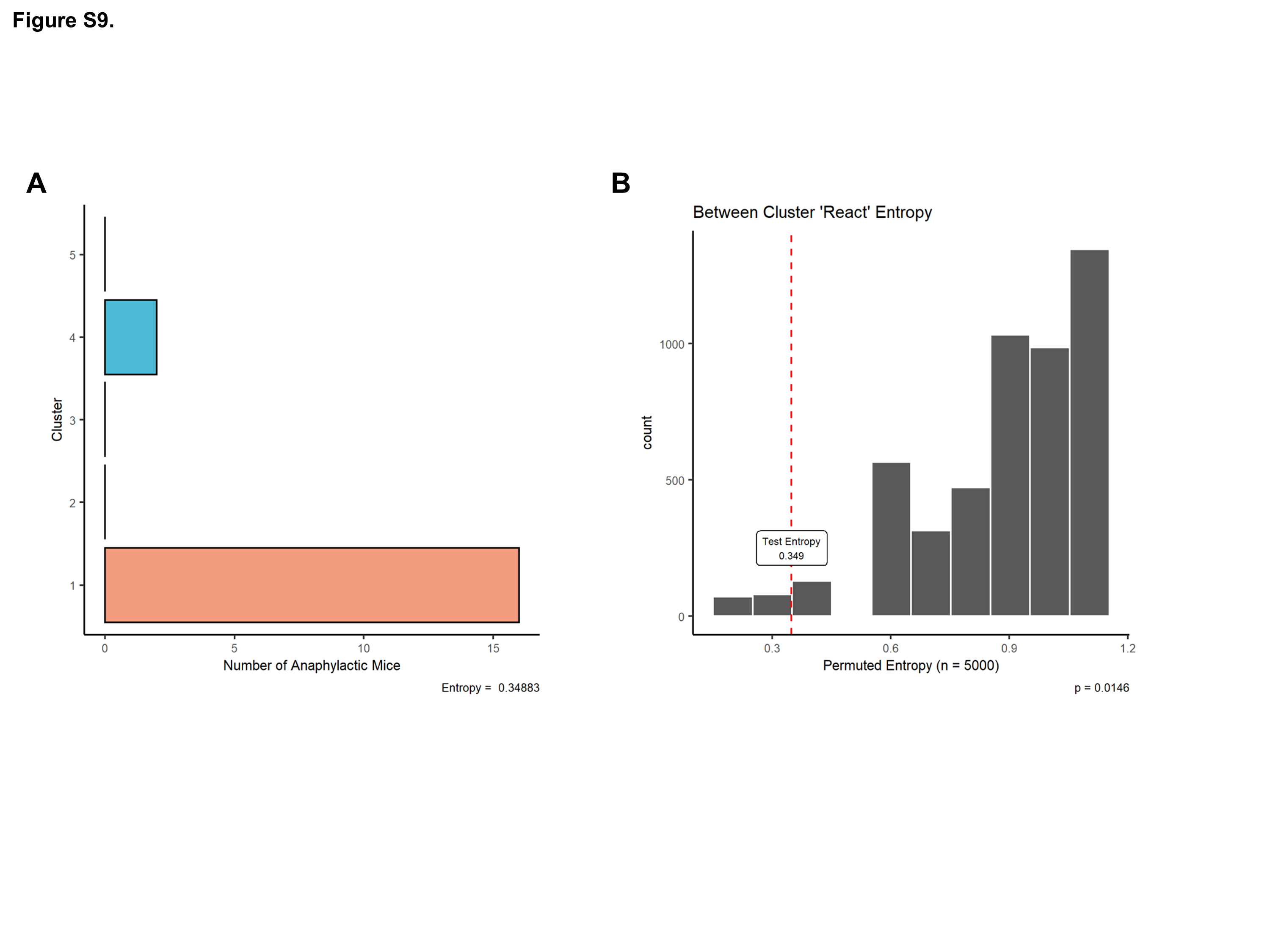

Supplement: Supplementary file 10 [file Image_9.tif]

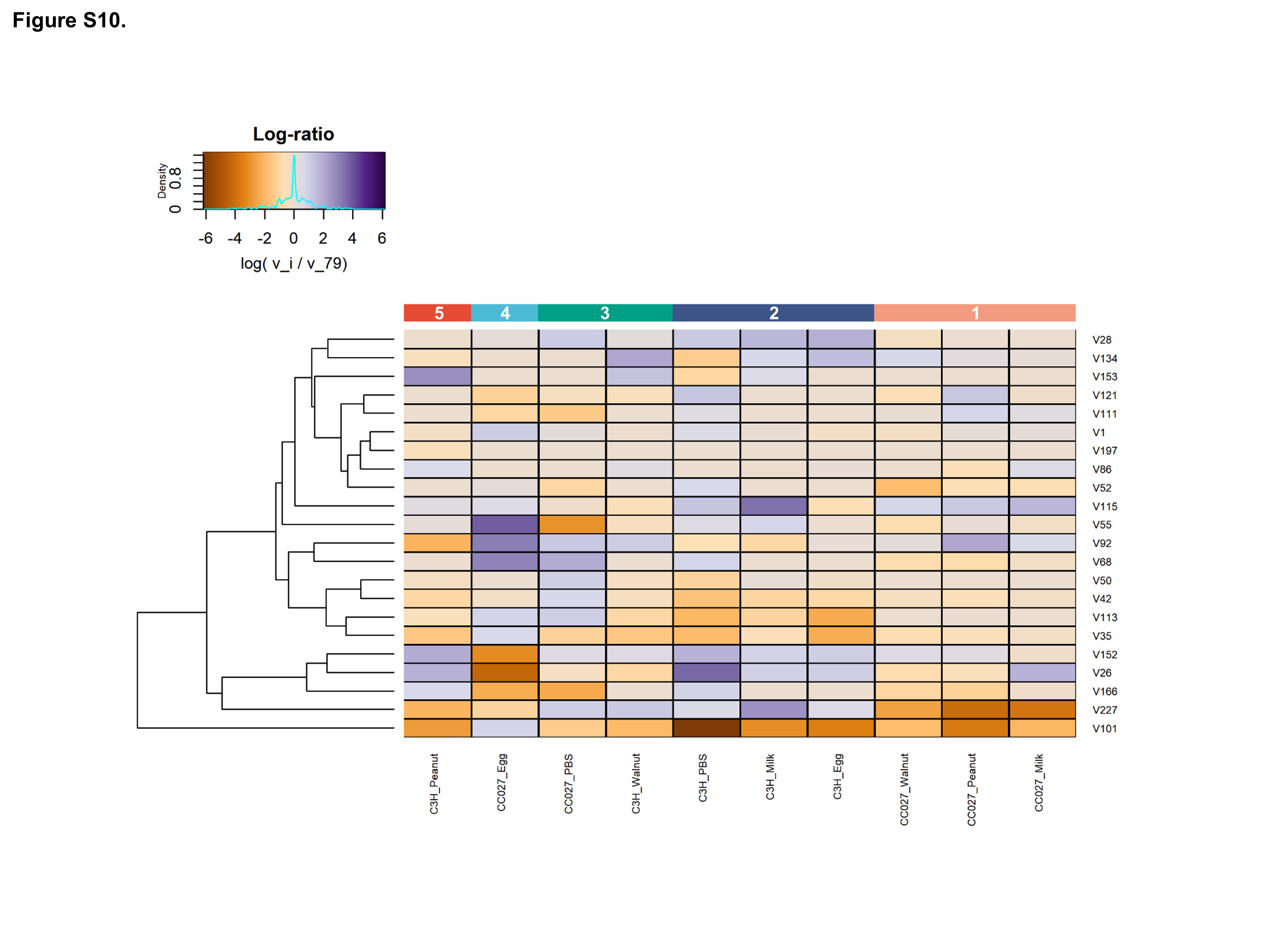

Supplement: Supplementary file 11 [file Image_10.tif]

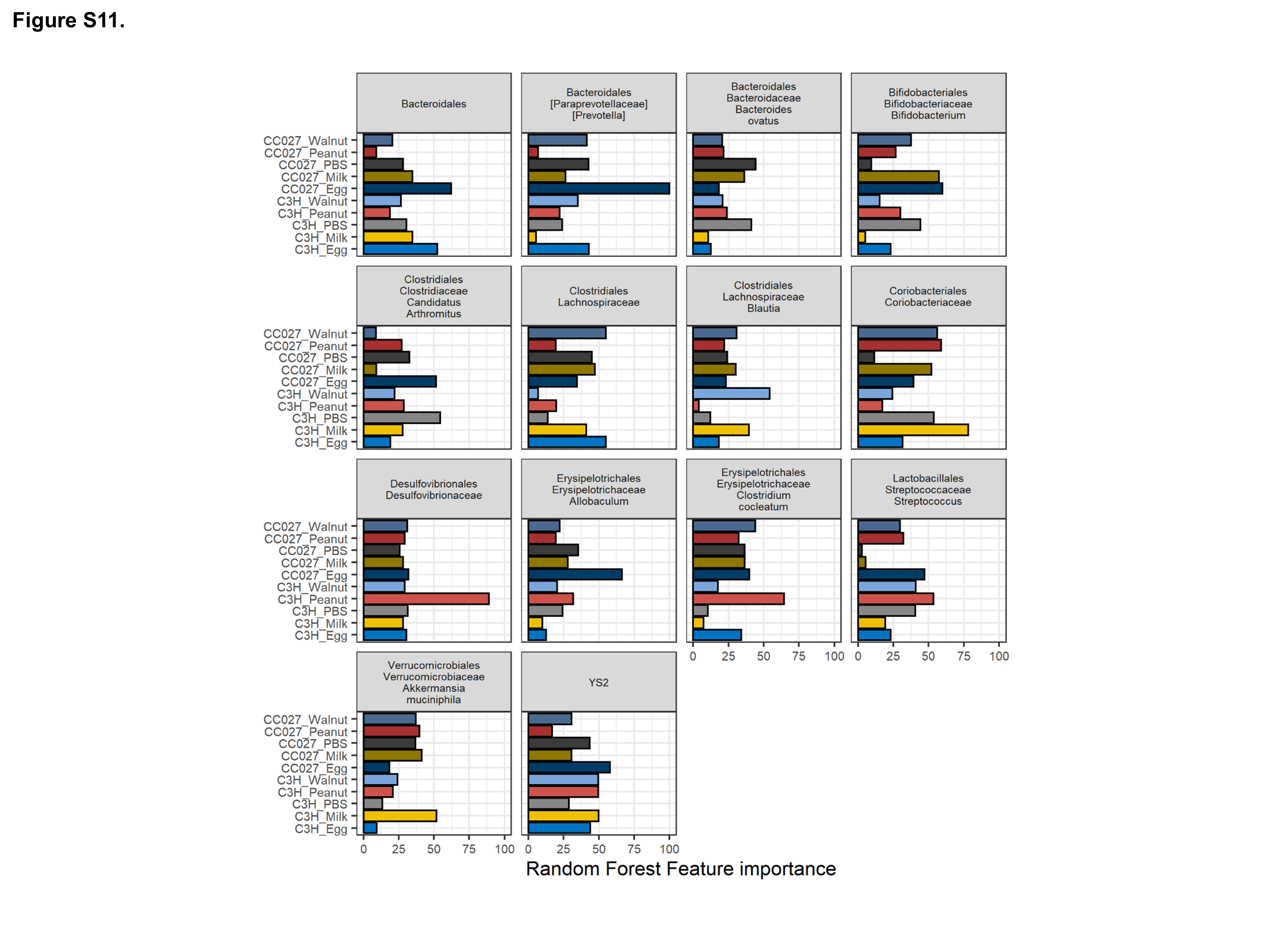

Supplement: Supplementary file 12 [file Image_11.tif]
